# Role of Arginase 2 in Systemic Metabolic Activity and Adipose Tissue Fatty Acid Metabolism in Diet-Induced Obese Mice

**DOI:** 10.3390/ijms20061462

**Published:** 2019-03-22

**Authors:** Reem T. Atawia, Haroldo A. Toque, Mohamed M. Meghil, Tyler W. Benson, Nicole K. H. Yiew, Christopher W. Cutler, Neal L. Weintraub, Ruth B. Caldwell, Robert W. Caldwell

**Affiliations:** 1 Department of Pharmacology and Toxicology, Medical College of Georgia, Augusta, GA 30912, USA; RATAWIA@augusta.edu (R.T.A.); HFLORESTOQUE@augusta.edu (H.A.T.); KYIEW@wustl.edu (N.K.H.Y.); 2 Vascular Biology Center, Medical College of Georgia, Augusta, GA 30912, USA; TBENSON@augusta.edu (T.W.B.); NWEINTRAUB@augusta.edu (N.L.W.); RCALDWEL@augusta.edu (R.B.C.); 3 Department of Periodontics, Dental College of Georgia, Augusta University, Augusta, GA 30912, USA; MMEGHIL@augusta.edu (M.M.M.); CHCUTLER@augusta.edu (C.W.C.); 4 Department of Oral Biology and Diagnostic Sciences, Dental College of Georgia, Augusta University, Augusta, GA 30912, USA; 5 Current address: Department of Pathology and Immunology, Washington University School of Medicine, St. Louis, MO 63110, USA; 6 Charlie Norwood VA Medical Center, Augusta, GA 30904, USA

**Keywords:** arginase, obesity, inflammation, metabolism, endothelial dysfunction, fatty acid oxidation, AMPK-α

## Abstract

Visceral adipose tissue (VAT) inflammation and metabolic dysregulation are key components of obesity-induced metabolic disease. Upregulated arginase, a ureahydrolase enzyme with two isoforms (A1-cytosolic and A2-mitochondrial), is implicated in pathologies associated with obesity and diabetes. This study examined A2 involvement in obesity-associated metabolic and vascular disorders. WT and globally deleted A2(^−/−^) or A1(^+/−^) mice were fed either a high fat/high sucrose (HFHS) diet or normal diet (ND) for 16 weeks. Increases in body and VAT weight of HFHS-fed WT mice were abrogated in A2^−/−^, but not A1^+/−^, mice. Additionally, A2^−/−^ HFHS-fed mice exhibited higher energy expenditure, lower blood glucose, and insulin levels compared to WT HFHS mice. VAT and adipocytes from WT HFHS fed mice showed greater A2 expression and adipocyte size and reduced expression of *PGC-1α, PPAR-γ,* and adiponectin. A2 deletion blunted these effects, increased levels of active *AMPK-α*, and upregulated genes involved in fatty acid metabolism. A2 deletion prevented HFHS-induced VAT collagen deposition and inflammation, which are involved in adipocyte metabolic dysfunction. Endothelium-dependent vasorelaxation, impaired by HFHS diet, was significantly preserved in A2^−/−^ mice, but more prominently maintained in A1^+/−^ mice. In summary, A2 is critically involved in HFHS-induced VAT inflammation and metabolic dysfunction.

## 1. Introduction

Obesity, particularly central obesity, is associated with chronic inflammation and is considered to be a key contributor to metabolic dysfunctions. It is also an independent risk factor for all-cause mortality, and is an especially high-risk factor for cardiovascular diseases [1,2,3,4]. Obesity is a state of excess nutrients that exceed the buffering capacity of adipocytes, the main component of adipose tissue [5]. Adipocytes thus become larger, hypoxic, and insulin resistant which results in hyperglycemia and pathological ectopic fat deposition [6].

These adipocytes also become senescent and undergo apoptosis, inducing a strong inflammatory response and fibrosis [7,8]. In addition, during obesity, adipocytes have an altered adipokine secretion profile in which pro-inflammatory adipokines are elevated and anti-inflammatory adipokines, such as adiponectin, are reduced further exacerbating this deleterious environment. These compounding effects lead to chronic inflammation, insulin resistance, and development of cardiovascular disease [9]. A possible strategy to reduce adiposity is to increase energy expenditure by inducing mitochondrial biogenesis and activating fatty acid oxidation in the visceral adipose tissue (VAT) [10]. This can be achieved by activating 5′ adenosine monophosphate-activated protein kinase-α (*AMPK-α*), the central regulator of energy homeostasis that promotes energy conservation and induces fatty acid β-oxidation [11].

Here we report on studies showing the involvement of the mitochondrial isoform of the arginase enzyme, arginase 2 (A2) in visceral adiposity and the associated inflammation and metabolic dysfunction. Arginase is a ubiquitous enzyme that has two isoforms, cytosolic A1 and mitochondrial A2 [12]. We, and others, have shown that increased arginase expression is involved in cardiovascular disease states in humans and animal models by competing with nitric oxide synthase (*NOS*) for their common substrate, L-arginine and limiting synthesis of nitric oxide (NO) [12,13,14]. Recent studies in animal models of diet-induced or genetic obesity also have shown that increased arginase expression is involved in vascular and adipose tissue inflammation [15,16]. Our laboratory recently demonstrated that mice challenged with a high fat/high sucrose diet (HFHS) were protected against obesity-induced vascular endothelial dysfunction and adipose tissue inflammation by the deletion of A1 in endothelial cells or by chronic treatment with an arginase inhibitor. This protection was associated with preserved levels of NO and reduced oxidative stress [17,18].

Previous studies have shown that the global deletion of A2 protects against age-associated pancreatic β-cell apoptosis and obesity-induced atherosclerosis and insulin resistance [19,20]. However, the precise role of A2 in systemic metabolic activity and adipocyte lipid metabolism during obese conditions remains unknown. In addition, the effect of A2 on the macrophage inflammatory response is controversial [16,21,22]. In this study, we examined the specific metabolic consequences of A2 deletion in a mouse model of obesity and investigated the underlying mechanisms behind these observations, as well as the role of A2 expression in the macrophage polarization phenotype in adipose tissue.

## 2. Results

### 2.1. A2 Deletion Prevents High Fat/High Sucrose (HFHS) Diet-Induced Obesity

**Figure 1:**
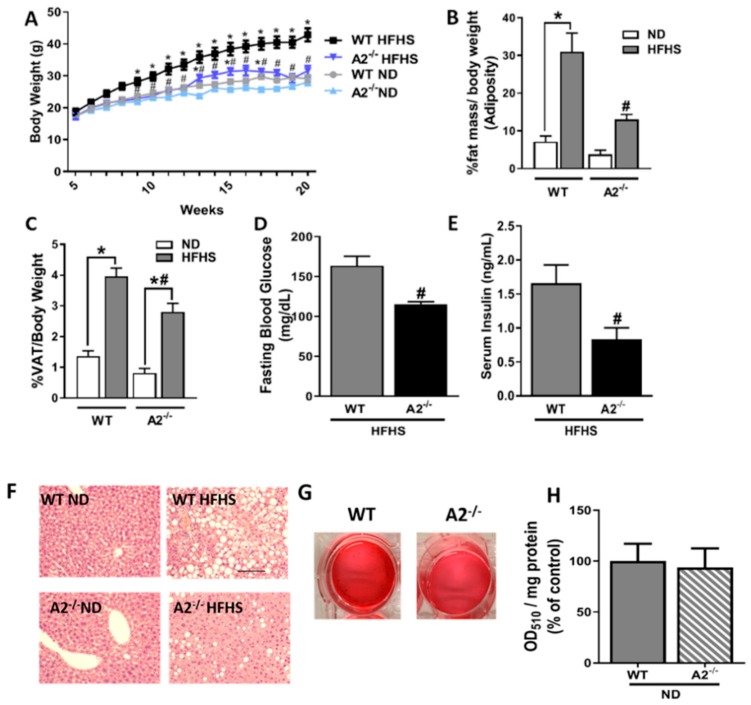
A2 deletion attenuates HFHS diet-induced increases in body weight, adiposity, VAT expansion, fasting blood glucose, postprandial serum insulin, and lipid accumulation in the liver without affecting adipocyte differentiation. Growth curves (**A**), n = 8–17 per group; percentage of body fat mass measured by nuclear magnetic resonance spectroscopy (**B**), n = 5–9 per group; percentage of VAT weight relative to body weight (**C**), n = 8–14 per group; fasting blood glucose (**D**) and fed serum insulin (**E**), n = 5–8 mice per group are shown for WT and A2^−/−^ mice. Values are represented as mean ± SEM. * *P* < 0.05 when compared to ND-fed mice within the same genotype, # *P* < 0.05 when compared to WT on the same diet. Representative photomicrographs of hemotoxylin and eosin-stained liver tissue sections from WT and A2^−/−^ after 16 weeks on HFHS or ND (**F**), scale bar = 50 μM. Representative images of cytoplasmic lipid droplets in preadipocytes from SVF of VAT of WT and A2^−/−^ mice stained with Oil Red O after eight days of *in vitro* differentiation (**G**) and the corresponding quantification (**H**), n = 3 per group).

Wild type (WT) male mice fed the HFHS diet for 16 weeks showed significant increases in body weight and total adiposity, as determined through nuclear magnetic resonance (NMR) analysis of the percentage of fat mass/body weight (Figure 1A,B, respectively). These increases in body weight and fat mass were largely prevented in the A2^−/−^ mice. The relative amount of VAT weight to body weight was also significantly increased in the WT mice fed the HFHS diet (Figure 1C). This alteration also was significantly attenuated in the A2^−/−^ mice. There were no differences in weight gain, fat mass percentage, or visceral adiposity between WT and A2^−/−^ mice fed the normal chow diet (ND).

A2^−/−^ HFHS-fed mice also showed significantly lower levels of fasting blood glucose and postprandial serum insulin when compared to WT HFHS-fed mice (Figure 1D,E, respectively). These results raised questions about the possible effect of A2 deletion on lipid accumulation in the liver or adipogenic differentiation. As shown in Figure 1F, liver sections from the WT HFHS group exhibited increased lipid deposits in the form of small vacuoles. This was ameliorated in the A2^−/−^ group. Additionally, there was no effect of A2 deletion on adipogenic differentiation as determined by Oil Red O staining for lipid accumulation in *in vitro* differentiated preadipocytes isolated from the stromal vascular fraction (SVF) of VAT (Figure 1G,H and Supplementary Figure S1).

### 2.2. A2 Deletion Ameliorates the HFHS Diet-Induced Decrease in Metabolic Rate and Energy Expenditure

**Figure 2:**
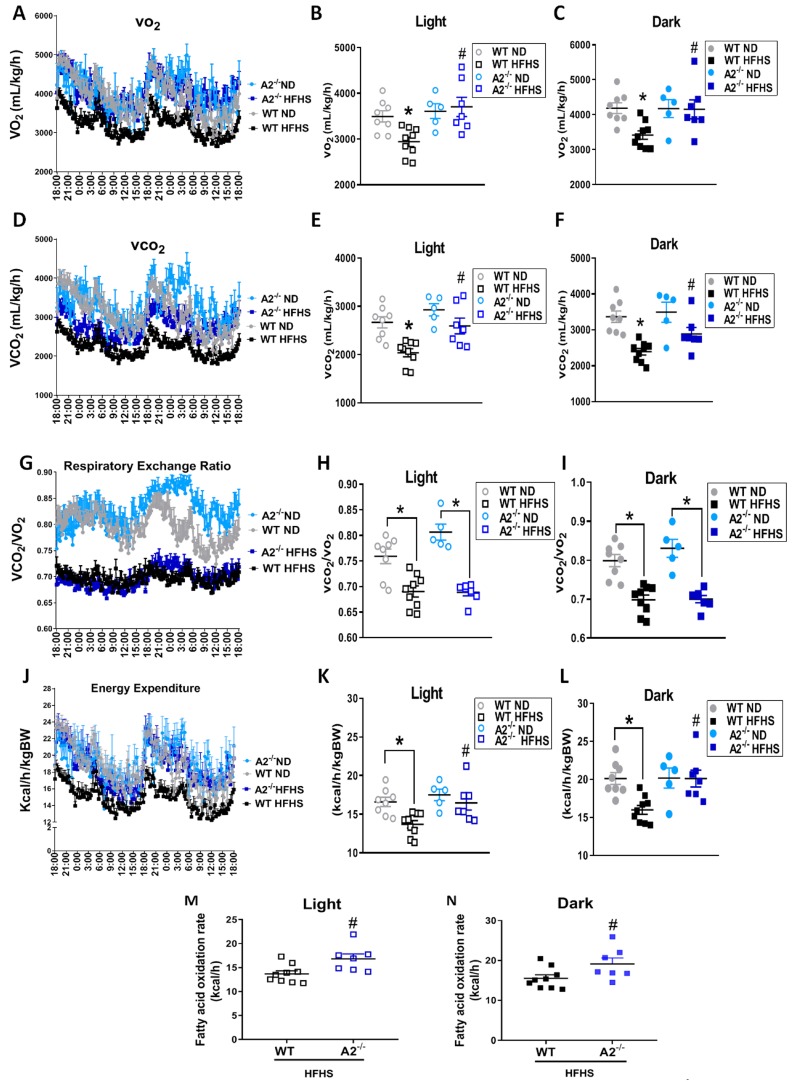
A2 deletion improves whole body metabolism. Oxygen consumption (VO_2_) (**A**–**C**) and carbon dioxide produced (VCO_2_) (**D**–**F**), represented as mL/kg/h; respiratory exchange ratio calculated as VCO_2_/VO_2_ (**G**–**I**) and energy expenditure (**J**–**L**) calculated as kcal of heat produced/h/body weight, fatty acid oxidation rate calculated as kcal/h during light and dark cycles (**M**,**N**). Values are means ± SEM; n = 5–9 mice/group. * *P* < 0.05 when compared to ND-fed mice within the same genotype, # *P* < 0.05 when compared to WT on the same diet.

To understand how A2 deletion contributes to metabolic activity and the leaner phenotype, metabolic cage studies were performed. Examination of quantity of food consumed (Supplementary Figure S2A) and locomotor activity (Supplementary Figure S2B) revealed that there were no significant differences among all the groups. However, the A2^−/−^ HFHS mice exhibited higher metabolic rates than WT HFHS mice, as evidenced by an increase in both O_2_ consumption (VO_2_) (Figure 2A–C) and CO_2_ release (VCO_2_) (Figure 2D–F) during both day/light and night/dark. The WT mice fed HFHS exhibited a significantly lower respiratory exchange ratio (VCO_2_/VO_2_) (Figure 2G–I) compared to those on ND, indicating their transition from carbohydrate to lipid metabolism. Due to the proportional increases in VO_2_ and VCO_2_ observed with A2^−/−^ HFHS mice compared to WT, there was no significant difference between WT and A2^−/−^ mice in the respiratory exchange ratio during day (Figure 2G,H) or night (Figure 2G,I), which indicates that A2 deletion has no effect on fuel preference. Interestingly, the HFHS diet-induced decrease in energy expenditure (heat produced) exhibited in WT mice was prevented by A2 deletion, during both day (Figure 2J,K) and night (Figure 2J,L). This effect could be attributed to the higher rates of fatty acid oxidation observed with the HFHS diet in A2^−/−^ versus WT mice (Figure 2M,N).

### 2.3. A2 Deletion Protected against HFHS Diet-Induced Adipocyte Hypertrophy and VAT Fibrosis

**Figure 3:**
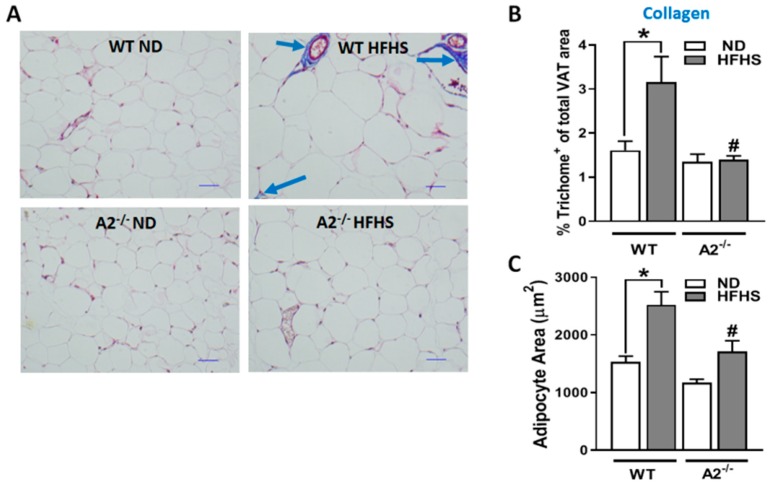
A2 deletion limits HFHS-induced VAT fibrosis and adipocyte hypertrophy. Representative photomicrographs of VAT show Masson’s trichrome staining of collagen/fibrosis depicted in blue color (**A**), Scale bar = 20 μM and quantified as % of trichrome positive area to total VAT area (**B**). Quantification of HFHS diet-induced increases in adipocyte area is shown for WT and A2^−/−^ mice (**C**). Data are presented as mean ± SEM, n = 3–5 mice/group. * *P* < 0.05 when compared to ND-fed mice within the same genotype, # *P* < 0.05 when compared to WT on the same diet.

We also examined the effects of A2 deletion on HFHS diet-induced adipocyte expansion and fibrosis of the extracellular matrix. Analysis of extracellular matrix fibrosis/collagen deposition by Masson’s trichrome staining showed a marked increase in collagen fibers (blue stain) in the VAT from the HFHS mice compared to the ND controls (Figure 3A). The A2 deletion completely blocked this HFHS-induced extracellular matrix fibrosis, as indicated by less blue staining compared to the VAT samples from WT mice on the HFHS diet (Figure 3B). Morphometric analysis of the adipocytes showed a significant increase in the size of the cells in the WT HFHS group compared to the ND control. This HFHS diet-induced hypertrophy was attenuated in the A2^−/−^ mice (Figure 3C).

### 2.4. A2 Deletion Protected against HFHS Diet-Induced Pro-Inflammatory Macrophage Infiltration in the VAT

**Figure 4:**
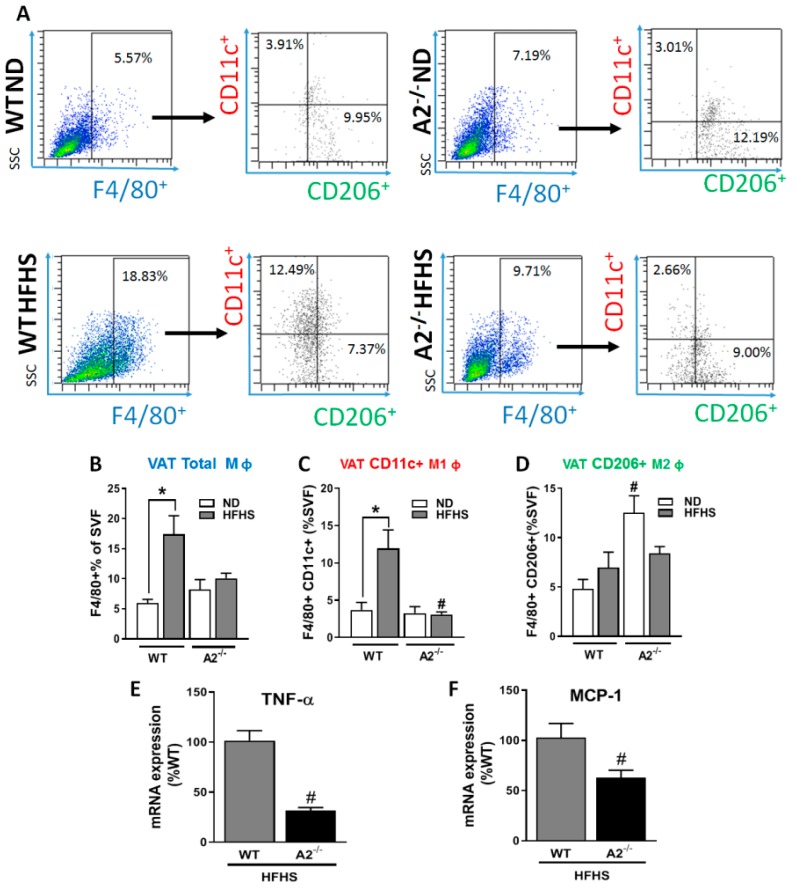
A2 deletion limits HFHS-induced increase in pro-inflammatory macrophages. Flow cytometry dot plots show F4/80^+^ SVF cells gated using PE channel against side scatter (SSC), subsets of F4/80^+^ cells were identified based on surface expression of CD11c and CD206 from WT and A2^−/−^ using unstained cells as negative control (**A**). Quantification of the plots shows the effect of A2 deletion on the percentage of F4/80^+^ cells (**B**), (F4/80^+^CD11c^+^) (**C**), and (F4/80^+^CD206^+^) (**D**) of SVF, representing total macrophage, pro-inflammatory M1-like and anti-inflammatory M2-like macrophages, respectively. Values are means ± SEM; n = 5–6 mice/group. Adipocyte mRNA expression of *TNF-α* (**E**) and *MCP-1* (**F**), n = 4 mice/group. * *P* < 0.05 when compared to ND-fed mice within the same genotype, # *P* < 0.05 when compared to WT on the same diet.

Inflammation is a late-stage consequence of pathological expansion and fibrosis of the VAT. Flow cytometric analysis of the VAT SVF showed over a 3-fold increase in macrophage infiltration in the VAT of the WT HFHS mice compared to ND mice (Figure 4A,B); an effect that was prevented in the A2^−/−^ animals. To determine the phenotype of the infiltrating macrophages, we used antibodies against the integrin CD11c, which is abundant in pro-inflammatory M1-like macrophages, and the mannose receptor CD206, which is abundant in anti-inflammatory M2-like macrophages [18]. The HFHS diet treatment resulted in a marked elevation in the number of pro-inflammatory M1-like infiltrating macrophages, which was abolished in the A2^−/−^ mice (Figure 4A,C). Interestingly, the A2^−/−^ mice fed the ND showed a significantly higher percentage of CD206-positive anti-inflammatory macrophages compared to the WT ND group (Figure 4A,D). Consistent with the lack of an increase in pro-inflammatory macrophage in the A2^−/−^ VAT after HFHS feeding, mRNA expression of the pro-inflammatory cytokine, tumor necrosis factor *(TNF)-α*, and chemokine, monocyte chemoattractant protein-1 *(MCP-1)* were lower in adipocytes from A2^−/−^ mice compared to those from WT mice (Figure 4E,F, respectively).

### 2.5. The HFHS Diet Increased A2 and Hypoxia-Inducible Factor (HIF)-1α Expressions in the VAT

**Figure 5:**
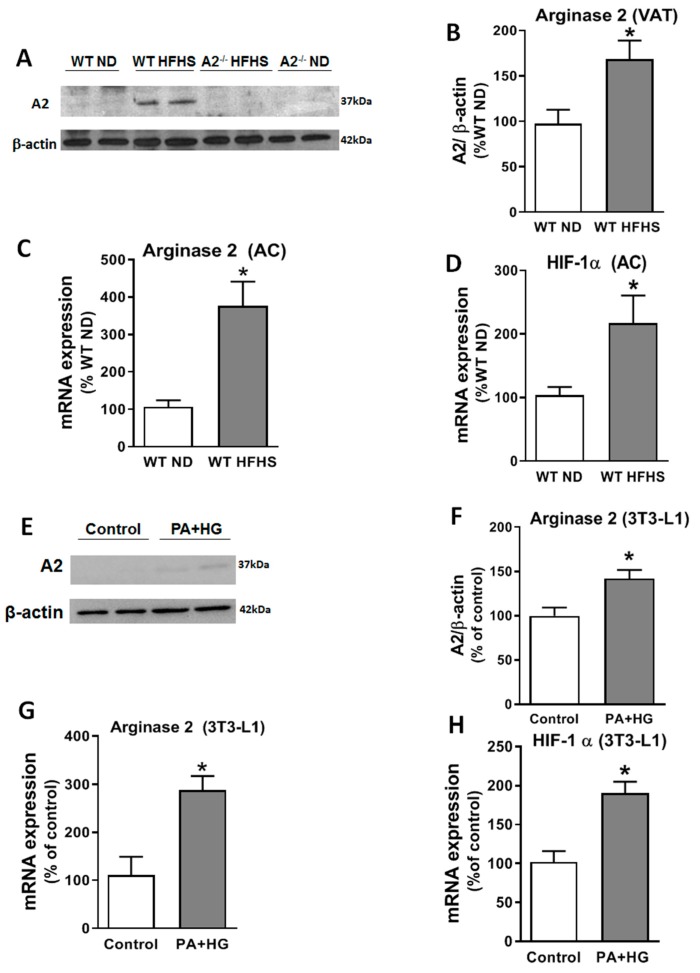
HFHS increases A2 expression in VAT and adipocytes. Representative western blot (**A**) and densitometry analysis (**B**) showing increased A2 protein levels in VAT of WT mice fed HFHS diet (n = 4 per group). qRT-PCR showing increased A2 (**C**) and HIF-1α (**D**) mRNA levels in mature adipocytes (AC) isolated from VAT of WT mice fed HFHS diet (n = 6–9 per group). Results are shown as a percentage of levels in WT mice fed ND. Representative western blot (**E**) and densitometry analysis (**F**) showing increased A2 protein levels in differentiated 3T3-L1 cell line treated with 250 μM of palmitate (PA) and high-glucose (HG) media (25 mM) for 7 days compared to normal glucose media (n = 4 per group). qRT-PCR showing increased A2 (**G**) and *HIF-1α* (**H**) mRNA levels in differentiated 3T3-L1 cells treated with PA/HG (n = 3–4 per group). Data are presented as mean ± SEM. * *P* < 0.05.

To further assess the role of A2 on the pathological expansion, fibrosis, and inflammation of the VAT, we evaluated A2 expression in VAT samples (by western blot) and in isolated adipocytes (by RT-PCR) from the HFHS and ND mice. These analyses showed a 72% elevation of A2 protein level in the VAT (Figure 5A,B) and a 277% increase in A2 mRNA levels in isolated adipocytes from WT HFHS-fed mice (Figure 5C). Additionally, we examined HIF-1α mRNA expression in isolated adipocytes because hypoxia in adipose tissue is common under obese conditions and has been shown to increase A2 expression in other cell types [23,24]. This analysis showed a significant upregulation of hypoxia-inducible factor *(HIF)-1α* mRNA in adipocytes from WT HFHS mice compared to the ND group (Figure 5D). In line with these results, we found that differentiated 3T3-L1 cells exposed to palmitic acid (250 µM) and high glucose (25 mM) (PA+HG) for seven days exhibited increased expression levels of A2 protein (Figure 5E,F) and mRNA (Figure 5G), as well as *HIF-1α* mRNA (Figure 5H) compared to cells maintained in control media. The A2 protein was not detected in western blots from A2^−/−^ mice. The levels of A1 mRNA in isolated adipocytes (Supplementary Figure S3) were not altered by A2 deletion.

### 2.6. A2 Deletion Enhanced Expression of Genes Involved in Fatty Acid β-Oxidation and Preserved Mitochondrial Density

**Figure 6:**
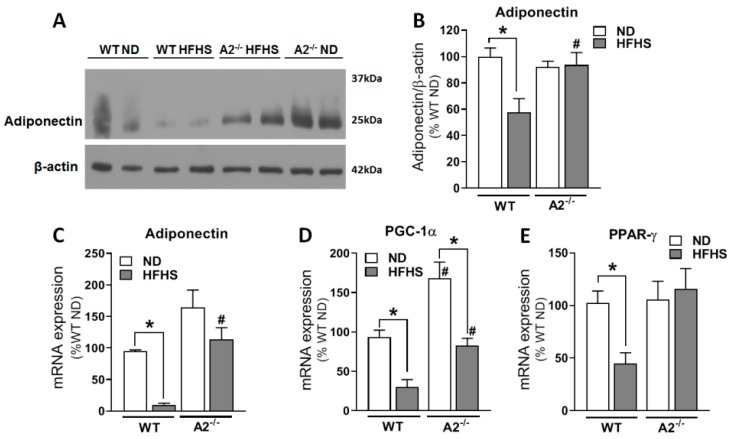

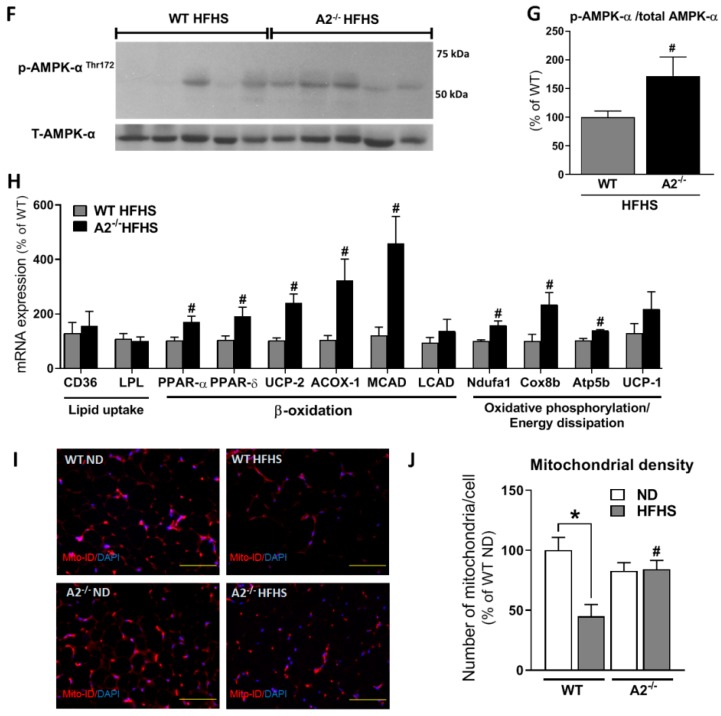
A2 deletion enhanced adipocyte expression of genes involved in fatty acid metabolism and preserved mitochondrial density. Representative western blot (**A**) and densitometry analysis normalized to the loading control (β-Actin) (**B**) showing adiponectin protein levels in VAT, n = 3–4/group. Adipocyte mRNA expression of adiponectin (**C**), n = 5–7/group. Adipocyte mRNA expression of *PGC-1α* (**D**) and *PPAR-γ* (**E**), n = 5–8 per group. Representative western blot (**F**) and densitometry analysis (**G**) showing ratio of *p-AMPK-α* to total-*AMPK-α* in VAT, n = 5–8/group and mRNA expression of genes involved in fatty acid uptake, β-oxidation and oxidative phosphorylation (OXPHOS), n = 4–6 (**H**). Immunofluorescence images of VAT sections stained with MitoID-Red as an estimate of mitochondrial mass (**I**) with quantitation (**J**); scale bar = 50 μM. Values are means ± SEM; * *P* < 0.05 when compared to ND-fed mice within the same genotype, # *P* < 0.05 when compared to WT on the same diet.

To further explore the possible mechanisms by which A2 deletion improves metabolic function, we analyzed effects of the A2 deletion on genes involved in fatty acid metabolism. Consumption of HFHS diet by WT mice significantly reduced the expression of the anti-inflammatory, insulin-sensitizing adipokine, adiponectin (Figure 6A–C), and decreased mRNA expression of peroxisome proliferator-activated receptor gamma (*PPAR-γ*) coactivator 1 α (*PGC1α*) (Figure 6D) and *PPAR-γ* (Figure 6E) in visceral adipose tissue. PGC1α and PPAR-γ are both prominent enhancers of mitochondrial activity and fatty acid metabolism [25,26]. Moreover, expression and activity of P*GC1α* are upregulated by *AMPK-α* activation [27,28], which we found to be increased with A2 deletion (Figure 6F,G).

The CLAMS analysis of metabolic activity revealed that the WT and A2^−/−^ mice fed HFHS diets were both primarily metabolizing lipids. However, the A2^−/−^ group exhibited a higher rate of fatty acid oxidation, a leaner phenotype, and higher energy expenditure. This observation prompted the investigation of the effect of A2 deletion on genes involved in fatty acid metabolism. Adipocytes from the A2^−/−^ mice on the HFHS diet exhibited a marked increase in mRNA expression of genes involved in fatty acid β-oxidation, oxidative phosphorylation (OXPHOS), and energy dissipation when compared to WT mice (Figure 6H and Supplementary Figure S4). However, there were no differences in the expression of genes involved in fatty acid uptake between WT and A2^−/−^ mice.

Given the beneficial effects of the A2 deletion on energy expenditure and the fact that A2 is localized to the mitochondria, we postulated that the beneficial effects of the A2 deletion under conditions of obesity may involve preservation of the mitochondria. Sections of VAT were treated with a non-membrane potential-dependent mitochondrial stain, MitoID-Red, to determine mitochondrial density. Compared to WT ND mice, WT HFHS mice showed a 57% reduction in mitochondrial density in VAT. Mitochondrial density was preserved in HFHS-fed mice lacking A2. (Figure 6I,J).

### 2.7. A2 Deletion Attenuated Circulating Oxidative Species and Preserved NO Levels and Vascular Function

**Figure 7:**
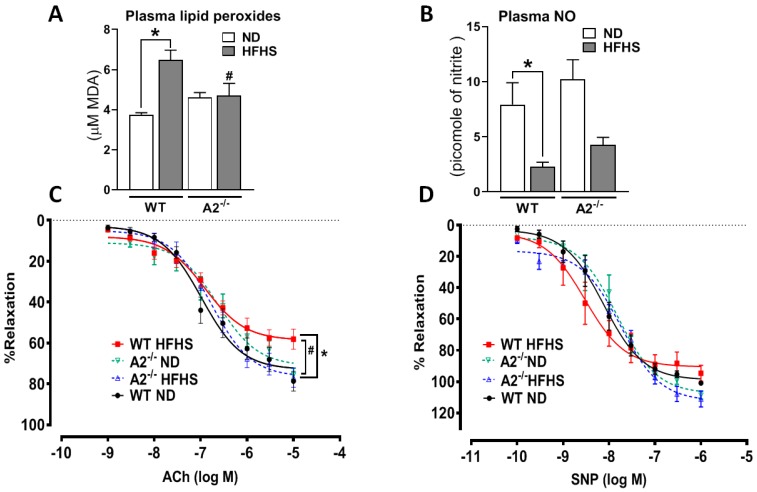
A2 deletion limited HFHS-induced oxidative stress, preserved NO levels and endothelium-dependent vasorelaxation to acetylcholine (ACh). Lipid peroxides are expressed as micromolar of malondialdehyde (MDA) (**A**), n = 5–9 mice/group, and plasma NO is expressed as picomoles of nitrite (**B**), n = 5–9 mice/group. Effects of A2 deletion on endothelium-dependent vasorelaxation to acetylcholine (ACh) (**C**), n = 7–10 mice per group, and sodium nitroprusside (SNP) (**D**), n = 4–5 mice per group. Values are mean ± SEM. * *P* < 0.05 when compared to ND-fed mice within the same genotype, # *P* < 0.05 when compared to WT on the same diet.

A2 deletion markedly attenuated HFHS-induced oxidative stress, as demonstrated by lower plasma lipid peroxide levels compared to the WT HFHS mice (Figure 7A). An optimal level of plasma NO has been proposed to be protective of endothelial and adipocyte function (24). Our analysis confirmed significantly lower plasma NO levels in WT mice on HFHS versus normal diet. This effect was blunted by A2 deletion (Figure 7B).

To determine the effect of excess nutrients and metabolic abnormalities on vascular function, we tested the vasodilation capacity of aortas in response to the endothelial-dependent vasodilator acetylcholine (ACh) and the endothelial-independent vasodilator sodium nitroprusside (SNP). Aortas from WT mice fed the HFHS diet exhibited reduced maximal vasorelaxation responses to ACh compared to the WT ND mice. Aortas from A2^−/−^ mice on HFHS diet showed maximal vasorelaxation responses similar to that of ND groups (Figure 7C). To confirm that the diet-induced vasorelaxant responses we observed were indeed endothelial-dependent, we tested the vasorelaxant responses of the aortas to the NO donor SNP. Our results showed similar responses across all groups (Figure 7D).

### 2.8. Arginase 1 Deletion Preserved Vascular Function in HFHS Fed Mice without Affecting the Gain in Body Weight

**Figure 8:**
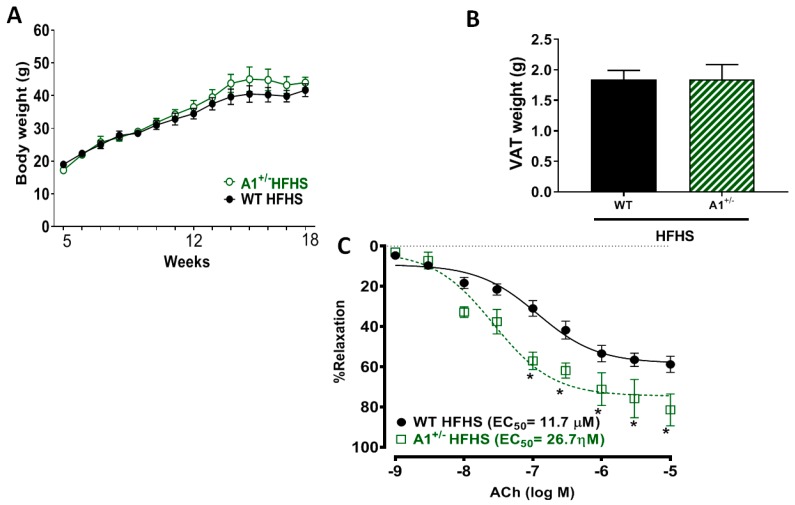
A1 deletion enhanced endothelium-dependent vasorelaxation with no effect on the gain of body and VAT weight. Effect of WT and A1 heterozygous deleted (A1^+/−^) mice challenged with HFHS diet on gain in body weight (**A**), VAT weights (**B**), and endothelium-dependent vasorelaxation to acetylcholine (ACh) (**C**). EC_50_: half maximal effective concentration for the ACh. Values are mean ± SEM, n = 4–6 mice/group. * *P* < 0.05 when compared to WT HFHS.

A1 heterozygous knockout (A1^+/−^) mice fed HFHS diet did not show any differences in gain of body weight (Figure 8A) or VAT weight (Figure 8B) compared to WT mice on the same diet. Interestingly, the global heterozygous deletion of arginase 1 (A1^+/−^) resulted in a pronounced aortic vasorelaxation with a marked increase in sensitivity to ACh (reduction in the EC_50_) as compared to responses of aorta from WT HFHS mice (Figure 8C).

### 2.9. Arginase Inhibition Prevented Vascular Endothelial Dysfunction Induced by Conditioned Medium (CM) from VAT of WT HFHS Mice

**Figure 9:**
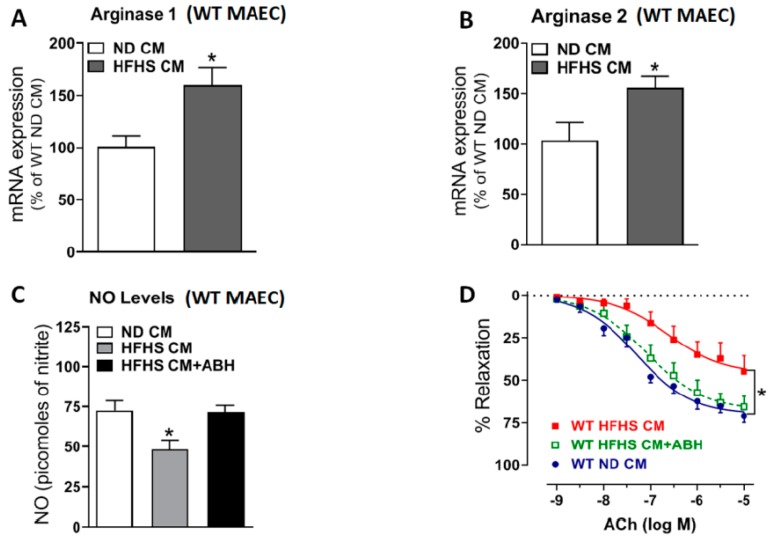
Vascular endothelial dysfunction induced by conditioned medium (CM) from VAT of WT-HFHS mice involves arginase upregulation. Conditioned media (CM) was prepared by incubating 50 mg VAT from either WT-ND or WT-HFHS mice for 24 h in M199 media. Presented are mRNA expression of A1 (**A**), A2 (**B**), and NO production (**C**) in MAEC. Additionally presented are endothelial-dependent vasorelaxant responses to acetylcholine in aortic rings from WT-ND mice treated for 24 h with CM from VAT of WT-ND and WT-HFHS mice in the presence/absence of ABH (100 μM) (**D**). Values are mean ± SEM. * *P* < 0.05 vs. WT-ND CM, # *P* < 0.05 vs. WT-HFHS CM (n = 4–5).

We were particularly interested in the effects of diet on endothelial cell function. Mouse aortic endothelial cells (MAEC) exposed to CM from VAT of WT HFHS mice (24 h) showed increased mRNA expression of A1 and A2 (Figure 9A,B) and reduced levels of NO (Figure 9C), compared to MAEC exposed to CM from WT ND mice. Aortic rings incubated with CM from WT HFHS mice for 24 h exhibited marked impairment of the endothelial-dependent vasorelaxant response to acetylcholine (ACh) (Figure 9D). Pretreatment with the arginase inhibitor ABH (100 μM) preserved NO levels and vasorelaxant responses to ACh (Figure 9C,D). This indicates the key role of arginase in obesity-induced endothelial dysfunction.

## 3. Discussion

Visceral (central) obesity is of particular importance as it directly correlates with metabolic and cardiovascular pathologies [1,2]. Our study found that A2 deletion ameliorated the diet-induced increase in body weight, whole-body adiposity, VAT weight, adipocyte hypertrophy, and insulin resistance. These effects were associated with decreases in adipose tissue inflammation, enhanced expression of genes involved in fatty acid metabolism and improved metabolic function. A previous study showed that mice fed high fat diet exhibited an increase in A2 expression in several tissues, including perivascular adipose tissue [29]. Treatment of mice on a high fat diet with an arginase inhibitor Nω-hydroxy-nor-L-arginine has been shown to prevent their gain of body and adipose tissue weight [30]. However, other obesity studies in mice lacking A2 only reported a trend toward less body weight gain compared to WT mice [16,31]. Reasons for this discrepancy are not clear. The age of mice when the diet begins and the duration of diet may affect the results. These studies, however, showed favorable outcomes in terms of insulin resistance and liver steatosis in A2^−/−^ mice, which are in line with our data. We found that A2^−/−^ mice on the HFHS diet had lower fasting blood glucose and postprandial serum insulin levels and less fat deposition in liver sections than WT mice on that diet. However, we did not observe an effect of A2 deletion on preadipocyte differentiation *in vitro*. This indicates that the leaner phenotype observed in A2^−/−^ mice may not be due to impaired adipocyte differentiation. In contrast, a recent study reported increased macrophage inflammation and hepatic steatosis in A2^−/−^ mice. The results may be confounded by a difference between the starting baseline weight of WT and A2^−/−^ mice [21].

A2 deletion contributes to an improved metabolic phenotype as shown by our metabolic cage studies in which A2^−/−^ mice on HFHS exhibited higher VO_2_, VCO_2_, energy expenditure and fatty acid oxidation rates compared to WT mice. Previous clinical and experimental studies have shown that obesity correlates with decreased energy expenditure, which leads to a net positive energy balance [32,33].

Obesity is characterized by pathological expansion of VAT in a locally hypoxic milieu [17,34]. This leads to immune cell infiltration, inflammation and adipose tissue fibrosis in both humans and rodents [34,35,36]. Fibrosis results in a rigid extracellular structure and mechanical stress on adipocytes, restraining their expansion [37]. In response to this stress, adipocytes release more inflammatory cytokines and chemokines, such as *TNF-α* and *MCP-1*, and less anti-inflammatory adipokines, like adiponectin [7,8]. The role of arginase in fibrosis is mediated in part via its conversion of L-arginine into L-ornithine, which is subsequently metabolized to L-proline, the major constituent of collagen [12]. In this study, our analyses of VAT showed that A2 deletion blunted the HFHS diet-induced collagen fiber deposition, prevented the infiltration of pro-inflammatory M1 macrophage and reduced adipocyte expression of *TNF-α* and *MCP-1*. Others have reported that deletion of A2 dampens the inflammatory macrophage response in HFHS fed mice as shown by a reduction in the inflammatory macrophages phenotype (M1) and expression of pro-inflammatory cytokines in adipose tissue [16]. Our study also sought to determine the effect of A2 deletion on the anti-inflammatory macrophage phenotype M2. Levels of the M2 macrophages were not altered by the HFHS diet; however, A2^−/−^ mice on ND showed significantly higher M2 levels compared to other groups. Initiation of M1 recruitment and/or polarization is well characterized and suggested to be caused by *TNF-α* secretion from hypertrophied adipocytes [38]. However, the initiator of the anti-inflammatory/resolving M2 macrophage recruitment/polarization in adipose tissues is not fully understood. Previous studies have shown increased as well as decreased M2 macrophage levels in obese adipose tissue [39,40].

The exact mechanism of obesity-induced A2 upregulation in VAT/adipocytes is not clearly understood. We hypothesized that hypoxic milieu in adipose tissue might play a critical role. In adipocytes, *HIF-1α* is considered the main mediator of the hypoxic response and involved in the pathophysiological consequences of adipocyte dysfunction during obesity [23]. It is reported that hypoxia also induces A2 upregulation in pulmonary artery smooth muscle [24]. In our study, the high fat diet increased the expression of *HIF-1α* and A2 in adipocytes from WT mice.

Reduced energy expenditure is due in part to downregulation of genes involved in fatty acid catabolism and oxidation [41]. While reduced fatty acid oxidation is considered to be a risk factor for obesity [42], it has been suggested that induction of energy expenditure during fatty acid oxidation in white adipose tissue reduces adiposity and promotes a lean phenotype [10]. Increased fatty acid β-oxidation in adipose tissue has been shown to reduce adiposity, inflammatory cell infiltration and improve insulin sensitivity [43,44,45]. A prior study showed mice lacking A2 were protected against high fat diet-induced steatohepatitis and that hepatic levels of active *AMPK-α* were higher than in WT mice [31]. Our study showed for the first time that A2 deletion increases activation of *AMPK-α* in adipose tissue. Active *AMPK-α* has been shown to induce expression and activity of *PGC-1α* [28], which coordinates the actions of *PPAR-γ* and *PPAR-α* to increase mitochondrial biogenesis and function, including expression of enzymes involved in mitochondrial fatty acid β-oxidation and oxidative phosphorylation [5,46]. Clinical and experimental studies have revealed decreased *PGC-1α* expression in fat depots of obese subjects [47]. *PPAR-γ* is considered the major positive regulator of adiponectin expression in adipose tissue [48]. Consistent with the protective effects of the A2 deletion in preserving *PGC-1α* and *PPAR-γ* expression in adipocytes, HFHS-fed A2^−/−^ mice showed significant preservation of adipocyte mitochondria as compared with the WT mice. Interestingly, our current findings also show that A2 deletion ameliorates the reduction of adiponectin expression, which is in line with a previous study [31]. It also improves adipocyte metabolic function under high fat diet challenge. Moreover, adiponectin has been shown to activate *AMPK-α* [49].

In patients with type 2 diabetes, obesity closely coexists with endothelial dysfunction [50], a hallmark of all cardiovascular diseases [51]. Several lines of evidence suggested a critical role of oxidative stress and increased lipid peroxidation in the link between obesity and vascular disorders [52,53]. Plasma levels of malondialdehyde (MDA) are a frequently used indicator of lipid peroxidation and have been shown to positively correlate with body mass index [54]. We found MDA elevated in WT mice on the HFHS diet, but not in mice lacking A2. Additionally, our group and others have shown that increased arginase expression/activity and a concomitant reduction in NO bioavailability are involved in obesity and diabetes-induced vascular dysfunction [17,55]. In this study, we found that A2 deletion blunted the HFHS-induced increase in plasma MDA and reduction in plasma NO levels. In addition, deletion of A2 prevented HFHS-induced vascular endothelial dysfunction. However, this effect was more pronounced in mice lacking A1, even though these mice had a similar weight gain as WT HFHS mice. This suggests a differential involvement of the two arginase isoforms in metabolic and vascular dysfunction associated with obesity as well as underscoring the critical role of A1 in vascular endothelial function which is in agreement with our recent reports [17,18]. It is possible that A2 deletion in HFHS-fed mice could indirectly improve VAT metabolic function by increasing vascular endothelial function and blood flow, rather than by direct effects on adipose tissue. However, it is unlikely, since vascular endothelial function for A1^+/−^ mice on that diet was markedly better and their gain in body/VAT weight was not affected.

We were also interested in determining the direct effects of factors released by VAT on endothelial cells and aortic explants without interference from systemic factors. We found that conditioned media from VAT of WT-HFHS mice increased expression of A1 and A2 in isolated endothelial cells in culture. It also reduced endothelial NO production and depressed endothelial-dependent vasorelaxant responses in isolated vessels. Both actions were prevented by arginase inhibition.

Collectively, our current study shows that deletion of A2 in mice alleviates HFHS-induced obesity and limits the associated metabolic dysfunctions. Our study demonstrates for the first time the beneficial effect of A2 deletion on energy expenditure and lipid metabolism. This may be attributed to increased *AMPK-α* activity, improved adipocyte metabolic function, suppression of adipose tissue inflammation and fibrosis. Additionally, there are a concomitant preservation of systemic NO levels and a normalized oxidative status, which can limit obesity-induced vascular dysfunction. Based on our findings, it appears important to examine the specific role of A2 in adipocytes and macrophages. Previous studies have linked A2 expression to insulin resistance, atherosclerosis and hepatic steatosis, which were attributed to an enhanced macrophage inflammatory response [16,31]. It has been shown that deletion of metabolically relevant genes, either in adipocytes or macrophages, yield similar metabolic phenotypes [56,57]. This study raises important questions: Are hypoxic conditions and elevated *HIF-1α* levels during obesity involved in A2 upregulation? Does A2 deletion reduce *HIF-1α* levels and thus mitigate adipocyte dysfunction? What is the interplay between endothelial cells and adipocyte dysfunction? And which one precedes the other? Also, what is the impact of A2 deletion in other tissues involved in metabolism, including muscle, liver and brown adipose tissue? Although the expression of *UCP-1* in VAT is low [58], A2 deletion showed a trend towards increased expression of this browning/beiging protein. Thus, the effect of A2 deletion on browning of adipose tissue warrant further investigation.

## 4. Materials and Methods

### 4.1. Animal Studies

All animal procedures were performed following the National Institutes of Health Guide for the Care and Use of Laboratory Animals and were approved on 05/26/2016 as protocol number 2010-0230 by the Institutional Animal Care and Use Committee at Augusta University. A2-deficient (A2^−/−^), A1 heterozygous-deficient (A1^+/−^), and wild type (WT) male mice bred from a C57BL/6J background were used in this study. The development, breeding, and genotyping of these mice have been previously described [59]. All animals developed to term, grew normally after birth and were fed a normal chow diet (ND) (calorie percentage: 18% fat, 24% protein, 58% carbohydrate with approximately 5% from sucrose, Harlan, Madison, WI, USA) or a high-fat high-sucrose (HFHS) diet (calorie percentage: 59% fat, 15% protein, 26% carbohydrate with 20% from sucrose, F#1850, BioServe, Frenchtown, NJ, USA). The diets were started four weeks after birth and continued for 16 weeks to experimentally mimic the conditions of metabolic syndrome [60,61]. Animals were kept at ambient temperature on a 12:12 h light/dark cycle and were fed ad libitum.

### 4.2. Body Weight, Body Composition, Fasting Blood Glucose, and Serum Insulin

Body weight was measured weekly throughout the experiment, and after 14 weeks on the diet, body composition was determined using a Bruker minispec LF90 TD-Nuclear magnetic resonance (NMR) analyzer. At the end of the study, the mice were fasted overnight and their fasting blood glucose (FBG) was measured using an AlphaTRAK glucometer. After determining their FBG, the mice were allowed access to food for 2 h [17]. Their post-prandial serum insulin levels were then determined using an Ultra-Sensitive Mouse Insulin ELISA Kit (Crystal Chem. USA, Downers Grove, IL, USA).

### 4.3. Indirect Calorimetry

After 14 weeks on the diets, an indirect respiration calorimetry system (CLAMS) was used to determine the metabolic rate and substrate utilization of the mice (Columbus Instruments, Columbus, OH, USA). Animals were maintained at 24 °C and a 12 h light/dark cycle. Food intake, respiratory exchange ratio (ratio of VCO_2_ to VO_2_), metabolic energy expenditure (heat produced), and locomotor activity were determined after a 24 h acclimatization period. The data were averaged over 48 h as previously described [62]. Rate of fatty acid oxidation was calculated according to the following formula: ([1.695 × VO_2_] − [1.701 × VCO_2_]) × 9/1000 [63].

### 4.4. Tissue Harvest

Mice were given an intraperitoneal (IP) injection of a ketamine/xylazine cocktail and exsanguinated. The blood, epididymal visceral adipose tissue (VAT), and liver were collected from each animal. A portion of the liver was fixed in 4% paraformaldehyde (PFA) for histological studies. The VAT was weighed and a portion was fixed for Masson-trichrome stain of collagen levels (fibrosis) and determination of adipocyte size using ImageJ software (NIH-Fiji, Bethesda, MD, USA) and Adiposoft plug-in (University of Navarra, Pamplona, Spain). Sections of the VAT were also used for immunofluorescence staining of the mitochondrial marker (MITO-ID^®^ Red, kit ENZ-51007, Enzo Life Sciences, Farmingdale, NY, USA) to assess mitochondrial density. Another portion of the VAT (500–700 mg) was fractionated using collagenase I (Worthington Biochemical Corporation, Lakewood, NJ, USA) to isolate floating mature adipocytes from the pelleted stromal vascular fraction (SVF) [18,64]. The remaining tissues were stored at −80 °C for protein analysis.

### 4.5. Flow Cytometry Analysis

The SVF was incubated with erythrocyte lysis buffer (Sigma-Aldrich; Darmstadt, Germany) for five minutes, washed with 1X D-PBS buffer and then centrifuged. 1 × 10^6^ SVF cells were re-suspended in ice-cold Flow Cytometry Staining Buffer (eBioscience, San Diego, CA, USA). A small portion of the cells was used to determine the percentage of live cells per sample with a hemocytometer based on trypan blue exclusion. Remaining cells were incubated in Fc Block (BD Bioscience, San Jose, CA, USA) for five minutes at 4 °C. Then, they were incubated with the following fluorochrome conjugated antibodies: phycoerythrin (PE)-conjugated anti-F4/80 (Cedarlane, Burlington, NC, USA), APC-conjugated anti-CD11c (BD Pharmingen™, San Diego, CA, USA), fluorescein isothiocyanate (FITC)–conjugated anti-CD206 (BD Pharmingen™) for 40 min. After incubation, the cell suspensions were washed and re-suspended in the flow cytometry staining buffer. The percentage of the total, M1, or M2 macrophages in the SVF were identified as being either F4/80^+^, F4/80^+^CD11c^+^, or F4/80^+^CD206^+^ double positive populations, respectively. Samples were acquired in MACSQuant^®^ Analyzer 10 and analyzed using MACSQuantify™ Software (Miltenyi Biotec Inc., Auburn, CA, USA).

### 4.6. Quantitative Reverse Transcription-PCR (Q-PCR)

Mature adipocytes isolated from the VAT were washed twice with PBS and the total RNA was isolated using TRIzol reagent (Invitrogen, Carlsbad, CA, USA) according to the manufacturer’s guidelines. Reverse transcription was performed using M-MLV reverse transcriptase (Invitrogen) to create cDNA. A StepONe Plus thermocycler (Applied Biosystems, Foster City, CA, USA) was used to amplify the cDNA with TaqMan Gene Expression assays for either A2 or Hypoxia-Inducible Factor *(HIF)-1α* (Applied Biosystems) or SYBR Green Dye (Applied Biosystems™) and specific primers for *TNF-α, MCP-1,* A1, *Adiponectin, PGC-1α, PPAR-γ, CD 36, LPL, PPAR-α, PPAR-δ, MCAD, LCAD, ACOX-1, UCP-2, COX-8b, Ndufa1, Atp5b, or UCP-1.* The primer sequences used are shown in Table 1. Relative gene expression was calculated using the 2^−∆∆*C*t^ method, the change in cycle threshold, determined by the initial increase in fluorescence above background, and normalized to hypoxanthine phosphoribosyl transferase (*HPRT*) expression as previously described [65,66]. PCR products were separated by electrophoresis on 1% agarose gels stained with ethidium bromide.

### 4.7. Adipose Tissue Western Blot

Visceral adipose tissue was collected and protein lysates were centrifuged at 100,000× g to remove neutral lipids. Then, 20 µg of the lysates were subjected to electrophoresis on SDS-polyacrylamide gels and transferred to nitrocellulose membranes where they were blocked in 5% non-fat milk (Bio-Rad, Hercules, CA, USA) and then incubated overnight at 4 °C with primary antibodies prepared in 2% bovine serum albumin. A2 (Santa Cruz Biotechnology cat. #Sc-20151, Dallas, TX, USA; 1:250), adiponectin (Santa Cruz Biotechnology cat. # Sc-136131, Dallas, TX, USA; 1:250), p-AMPK-α (Cell Signaling Technology cat. #2535 Danvers, MA, USA; 1:500), total AMPK-α (Cell Signaling Technology cat. #2603, Danvers, MA, USA; 1:500) and β-actin (Sigma-Aldrich cat. #A1978, 1:5000). The following day, membranes were washed three times in TBS-T (Tris-buffered saline with 0.5% Tween-20) and then incubated with the corresponding horseradish peroxidase-conjugated secondary antibodies (GE Healthcare, Piscataway, NJ, USA) for one hour at room temperature (1:2500). Signals were detected using an enhanced chemiluminescence system (GE Healthcare Bio-Science Corp., Piscataway, NJ, USA) and quantified by densitometry using ImageJ software and normalized to the loading control.

### 4.8. Preadipocyte Isolation and Adipogenic Differentiation In Vitro

The epididymal VAT was dissected from WT and A2^−/−^ mice and digested to isolate SVF pellets as described earlier. Cells were re-suspended, cultured, and grown in preadipocyte medium (Cell Applications, San Diego, CA, USA) [67], cells were resuspended, cultured and grown in preadipocyte medium (Cell Applications). Cells were passaged twice, and upon confluency, differentiated using adipocyte differentiation media (Cell Applications) for 2–3 days; then, the media was changed to adipocyte maintenance media (Cell Applications). After eight days of the differentiation process, cells were either collected in RIPA lysis buffer for protein quantification using Bio-Rad protein assay kit (Lowry method) or washed in PBS, fixed in 4% PFA (Thermofisher, Waltham, MA, USA) and stained with Oil Red O (Sigma) in 2-propanol. The stain was then extracted using 2-propanol and the color intensity was measured spectrophotometrically at 510 nm. The data is represented as OD units per mg protein [64]. The murine 3T3-L1 preadipocytes cell line was obtained from the ATCC, cultured in preadipocyte medium (ZenBio, Research Triangle Park, NC, USA) and subsequently differentiated in adipocyte differentiation medium (ZenBio) according to the manufacturer instructions. After seven days of differentiation, cells were exposed to the saturated free fatty acid, palmitate (16:0) (250 µM) (Sigma), and high glucose (25 mM) (PA/HG), a condition that mimics obesity *in vitro* or control media (no palmitate and 5mM of glucose) [68] or control media (no palmitate and 5mM of glucose). Cells were collected in TRIzol for PCR or in RIPA buffer for protein analyses.

### 4.9. Determination of Plasma Lipid Peroxide Levels and NO

Plasma levels of lipid peroxides were measured as an indicator of reactive oxygen species by assessing levels of thiobarbituric acid reactive substances spectrophotometrically at 532 and expressed as micromolar of malondialdehyde (MDA) [69]. NO production was determined using a Sievers 280i NO Analyzer (Boulder, CO, USA) and nitrite (NO_2_^−^) levels, the stable breakdown product of NO were quantified by a chemiluminescence detector [17].

### 4.10. Vascular Function

Vascular function was measured as previously described [17]. The aortas were rapidly excised and placed in cold Krebs buffer (pH 7.4). After removal of the perivascular fat, 2 mm rings were sectioned from thoracic aorta. Rings were then mounted and equilibrated in myograph baths (Danish MyoTechnology, Gainesville, FL, USA) filled with Krebs solution at 37 °C under a resting tension of 5.0 mN and perfused with 95% O_2_ and 5% CO_2_. A Power Lab system (AD Instruments, Colorado Springs, CO, USA) was used to measure the isometric force. The contraction in response to KCl (8 mmol/L) was assessed. After, the cumulative concentration-response curves to acetylcholine (ACh, an endothelium-dependent vasodilator, 0.001–10 μmol/L) or sodium nitroprusside (SNP, an endothelium-independent vasodilator, 0.0001–1 μmol/L) were obtained after pre-contracting the aortas with phenylephrine (1 μmol/L).

### 4.11. Conditioned Media (CM) Preparation from VAT and Endothelial Cell Treatment

To study the specific role of central obesity on endothelial function, conditioned media (CM) was prepared by incubating 50 mg of VAT from either WT ND or WT HFHS mice for 24 h in M199 media [70,71]. Mouse aortic endothelial cells (MAEC) were cultured, passaged as described [18] and exposed to CM for 24 h. In some of the experiments, cells were pretreated with the arginase inhibitor, 2-(S)-amino- 6-boronohexanoic acid (ABH), kindly provided by Corridor Pharmaceuticals, Inc. (Baltimore, MD, USA). In these experiments, the collected supernatant was used to measure NO levels and cells were collected for mRNA expression analysis of A1 and A2 using TaqMan Gene Expression assays. Additionally, aortic rings from WT ND mice were incubated with CM for 24 h *ex vivo* in the presence or absence of ABH, as previously described in [17] and vascular function was assessed.

### 4.12. Statistical Analysis

Results are expressed as mean ± SEM. Statistical analyses were tested by GraphPad Prism 7 (GraphPad Software Inc., La Jolla, CA, USA) using the unpaired Student’s two-tailed t-test or the two-way ANOVA followed by Tukey’s post hoc tests as appropriate. The effect of dietary intervention on body weight and vascular function were analyzed by two way repeated-measures ANOVA. Values of *P* < 0.05 were considered significant.

## Figures and Tables

**Table 1:** Sequence of primers used for RT-PCR experiments.

Mouse Gene Symbol	Gene Name	Forward	Reverse
*TNF-α*	Tumor necrosis factor alpha	GCACCACCATCAAGGACTCA	TCGAGGCTCCAGTGAATTCG
*MCP-1*	Monocyte chemoattractant protein 1	GGCTCAGCCAGATGCAGTTAA	CCTACTCATTGGGATCATCTTGCT
A 1	Arginase 1	TTGGGTGGATGCTCACACTG	TTGCCCATGCAGATTCCC
*Adiponectin*	Adiponectin	AAGGACAAGGCCGTTCTCT	TATGGGTAGTTGCAGTCAGTTGG
*PGC-1α*	Peroxisome proliferator-activated receptor gamma coactivator 1-alpha	AACCACACCCACAGGATCAGA	TCTTCGCTTTATTGCTCCATGA
*PPAR-ɣ*	Peroxisome proliferator-activated receptor gamma	CAAGAATACCAAAGTGCGATCAA	GAGCTGGGTCTTTTCAGAATAATAAG
*CD36*	Cluster of differentiation 36	TTGTACCTATACTGTGGCTAAATGAGA	CTTGTGTTTTGAACATTTCTGCTT
*LPL*	Lipoprotein lipase	CTGCTGGCGTAGCAGGAAGT	GCTGGAAAGTGCCTCCATTG
*PPAR-α*	Peroxisome Proliferator Activated Receptor alpha	GCGTACGGCAATGGCTTTAT	ACAGAACGGCTTCCTCAGGTT
*PPAR-δ*	Peroxisome Proliferator Activated Receptor Delta	CCTCGGGCTTCCACTACG	CACTTGTTGCGGTTCTTCTTC
*Ucp-2*	uncoupling protein 2	GCCCGGGCTGGTGGTGGTC	CCCCGAAGGCAGAAGTGAAGTGG
*Acox-1*	Acyl-CoA oxidase *1*	GCCAAGGCGACCTGAGTGAGC	ACCGCAAGCCATCCGACATTC
*MCAD*	Medium-chain acyl-CoA dehydrogenase	AACACTTACTATGCCTCGATTGCA	CCATAGCCTCCGAAAATCTGAA
*LCAD*	Long-chain acyl-CoA dehydrogenase	ATGGCAAAATACTGGGCATC	TCTTGCGATCAGCTCTTTCA
*Ndufa1*	NADH:Ubiquinone Oxidoreductase Subunit A1	ACATCCACAAATTCACCAACGG	AGCGATTGACTCCAGAGATACG
*COX-8b*	Cytochrome c oxidase subunit 8B	GAACCATGAAGCCAACGACT	GCGAAGTTCACAGTGGTTCC
*Atp5b*	ATP Synthase F1 Subunit Beta	CATTGGTGATGGTATTGCGC	TCCCAAACACGACAACTCC
*UCP-1*	Uncoupling protein 1	TCTTCTCAGCCGGAGTTTCAGCTT	ACCTTGGATCTGAAGGCGGACTTT
*HPRT*	Hypoxanthine Phosphoribosyltransferase 1	GAAAGACTTGCTCGAGATGTCATG	CACACAGAGGGCCACAATGT

## Supplementary Materials

The following are available online at https://www.mdpi.com/1422-0067/20/6/1462/s1. Supplementary Figures S1–S4 and their legends are presented separately.
