# Supplementary material for: Role of Arginase 2 in Systemic Metabolic Activity and Adipose Tissue Fatty Acid Metabolism in Diet-Induced Obese Mice
Source: Int J Mol Sci. 2019 Mar 22;20(6):1462. doi: 10.3390/ijms20061462 (PMC6472154; doi:10.3390/ijms20061462)
Supplement: Supplementary file 1 [file ijms-20-01462-s001.pdf]

Role of arginase 2 in systemic metabolic activity and adipose tissue fatty acid metabolism in diet-induced obese mice

Reem T. Atawia[1], Haroldo A. Toque[1,2], Mohamed M. Meghil[3], Tyler W. Benson[2], Nicole K. H. Yiew[1,2,5], Christopher W. Cutler[3], Neal L.Weintraub[2], Ruth B. Caldwell[2,4] and Robert W. Caldwell[1*]

Supplementary Materials

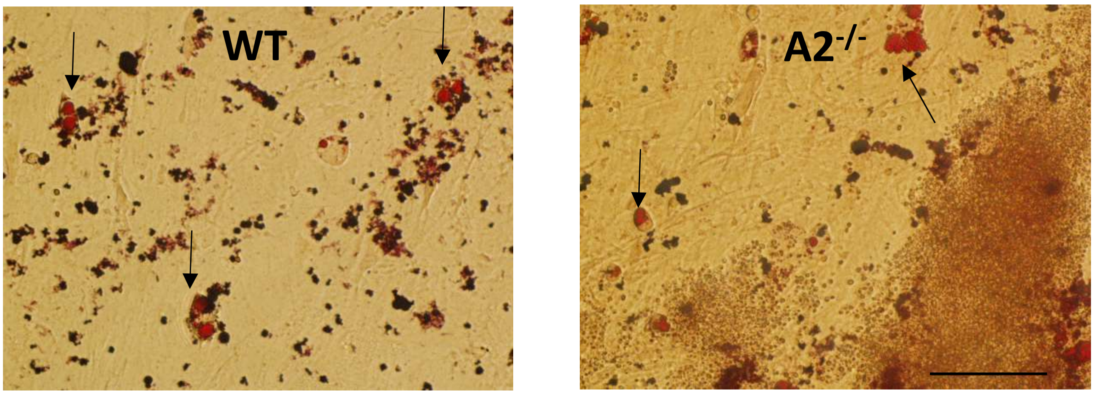

Supplementary Figure S1. Representative Images of cytoplasmic lipid droplets stained with Oil Red O after in vitro differentiation of preadipocytes from SVF of VAT,), scale bar=50 µm

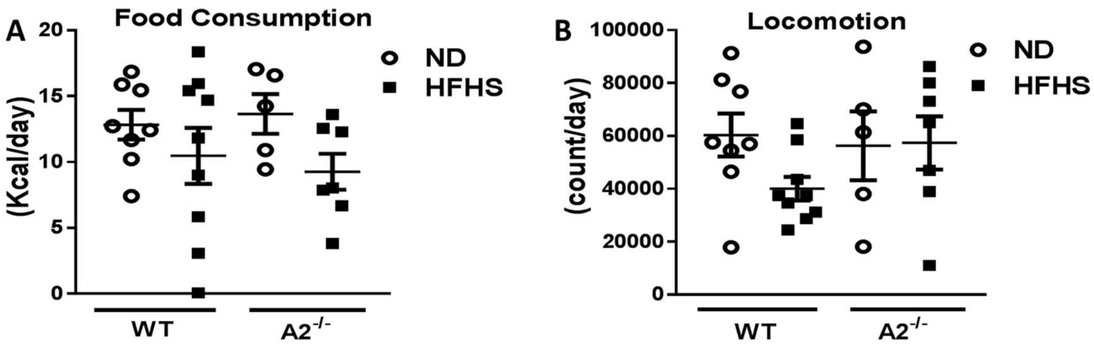

Supplementary Figure S2. Effect of HFHS on food intake calculated as kcal of food consumed/day (A) and locomotor activity calculated as steps/day (B) in WT and $A2^{-/-}$ mice. Values are means ± SEM, n=5-9 per group.

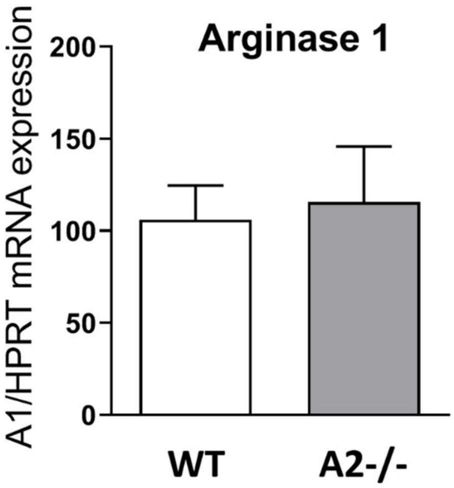

Supplementary Figure S3. Arginase 1 (A1) mRNA expression levels in adipocytes isolated from WT mice and mice globally lacking arginase 2 (A2-/-) fed HFHS diet for 16 weeks, determined by RT-PCR and normalized to hypoxanthine phosphoribosyl transferase (HPRT) expression. Data are presented as means ± SEM, n =6-7 per group.

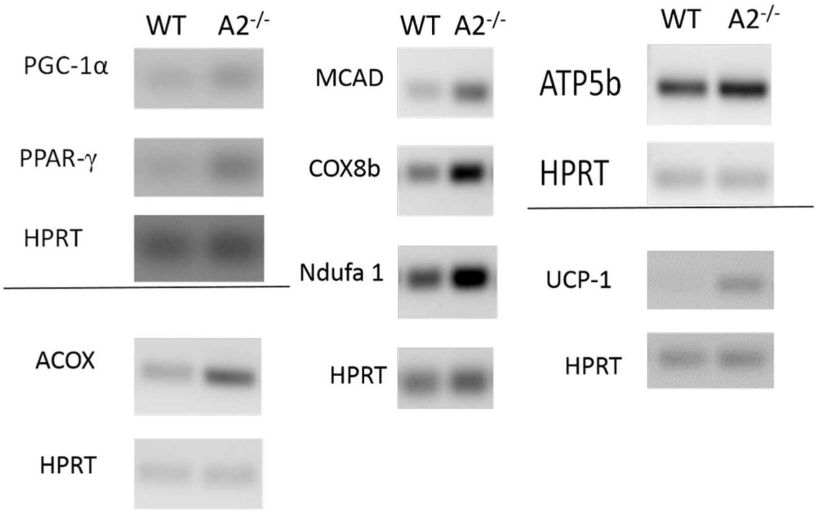

Supplementary Figure S4. Representative agarose gel electrophoresis of RT-PCR products of genes involved in fatty acid oxidation in adipocytes isolated from WT mice and mice globally lacking arginase 2 (A2-/-) on high fat-high sucrose (HFHS) for 16 weeks and normalized to hypoxanthine phosphoribosyl transferase (HPRT) expression
